# The treatment effect of endovascular therapy for chronic limb‐threatening ischemia with systemic sclerosis

**DOI:** 10.1111/1346-8138.17334

**Published:** 2024-06-19

**Authors:** Yoshihiro Matsuda, Tomoko Miyake, Hironobu Toda, Kota Tachibana, Hayato Nomura, Yoji Hirai, Yoshio Kawakami, Naoya Sakoda, Shin Morizane

**Affiliations:** ^1^ Department of Dermatology Okayama University Graduate School of Medicine, Dentistry and Pharmaceutical Sciences Okayama Japan; ^2^ Department of Cardiovascular Medicine Okayama University Graduate School of Medicine, Dentistry and Pharmaceutical Sciences Okayama Japan; ^3^ Department of Cardiovascular Surgery Okayama University Graduate School of Medicine, Dentistry and Pharmaceutical Sciences Okayama Japan

**Keywords:** chronic limb‐threatening ischemia (CLTI), endovascular therapy (EVT), revascularization, systemic sclerosis (SSc)

## Abstract

Systemic sclerosis (SSc) is a collagen disease with immune abnormalities, vasculopathy, and fibrosis. Ca blockers and prostaglandins are used to treat peripheral circulatory disturbances. Chronic limb‐threatening ischemia (CLTI) is a disease characterized by extremity ulcers, necrosis, and pain due to limb ischemia. Since only a few patients present with coexistence of CLTI and SSc, the treatment outcomes of revascularization in these cases are unknown. In this study, we evaluated the clinical characteristics and treatment outcomes of seven patients with CLTI and SSc, and 35 patients with uncomplicated CLTI who were hospitalized from 2012 to 2022. A higher proportion of patients with uncomplicated CLTI had diabetes and male. There were no significant differences in the age at which ischemic ulceration occurred, other comorbidities, or in treatments, including antimicrobial agents, revascularization and amputation, improvement of pain, and the survival time from ulcer onset between the two subgroups. EVT or amputation was performed in six or two of the seven patients with CLTI and SSc, respectively. Among those who underwent EVT, 33% (2/6) achieved epithelialization and 67% (4/6) experienced pain relief. These results suggest that the revascularization in cases with CLTI and SSc should consider factors such as infection and general condition, since revascularization improve the pain of these patients.

## 1 INTRODUCTION

Systemic sclerosis (SSc) is a collagen disease characterized by immune system abnormalities, vasculopathy, and fibrosis. Patients with SSc experience intense chronic pain.^1^, ^2^ In severe cases of SSc with infectious ulcers or severe tissue damage, amputation might be necessary to control these.^1^

Chronic limb‐threatening ischemia (CLTI) is a disease characterized by extremity ulcers, necrosis, and pain due to limb ischemia.^3^ Bohelay et al. reported that 21 of 45 (47%) patients with SSc with leg ulcers presented with ischemic lesions, and approximately one‐third of them had macrovascular lesions. Among the seven patients with macrovascular lesion, four (57%) underwent amputation.^4^ However, it is currently unclear whether patients with CLTI and SSc should be considered for revascularization, such as endovascular therapy (EVT) and bypass surgery. Here, we analyzed the clinical characteristics, outcomes, and prognosis of patients with CLTI and SSc.

## 2 METHODS

This study was approved by the Ethics Committee of Okayama University Hospital (No. 2206–043) and was performed in accordance with the 1975 Declaration of Helsinki. Consent for the publication of patient information was obtained by the authors from April 2012 to August 2022, 42 patients with CLTI were admitted to our hospital, including seven with CLTI and SSc and 35 with uncomplicated CLTI. We analyzed the characteristics, including onset ages, sex, and complications, and the differences in treatment outcomes and follow‐up time between the patients with CLTI, with and without SSc, using SPSS for Windows version 20.0. For univariate analyses, a one‐sided Fisher's exact test was used to compare categorical variables, and the Mann–Whitney U test was used to compare quantitative variables. The Kaplan–Meier method and log‐rank test were used for survival analysis. In all the analyses, *P* < 0.05 was considered statistically significant.

## 3 RESULTS

The median onset ages and sex of the CLTI and SSc and uncomplicated CLTI groups were 70 years (one male and six females) and 74 years (22 males and 13 females), respectively. There were no significant differences in onset age between the two groups, although a higher proportion of patients with uncomplicated CLTI had diabetes and male‐to female ratio compared to cases with CLTI and SSc (*P* = 0.012 and *P* = 0.018). Uncomplicated CLTI exhibited a trend toward a higher prevalence of chronic renal failure (CRF) (*P* = 0.053). There were no significant differences in the prevalence of smoking history, hypertension, dyslipidemia, and heart failure. Six of seven patients with CLTI and SSc, and 32 of 35 with uncomplicated CLTI underwent revascularization. Two of seven with CLTI and SSc, and 13 of 35 with uncomplicated CLTI underwent amputation. There were no significant differences in infection requiring antimicrobial therapy, or in the indications for revascularization or amputation (Table 1). Following revascularization, pain improved in four of six (66.7%) patients with CLTI and SSc, and in 21 of 32 patients (66.0%) with uncomplicated CLTI. There was no difference in pain improvement treated by revascularization between the two groups (*P* = 0.96).

**TABLE 1:** Background characteristics and treatment outcomes between cases with CLTI and SSc and those with uncomplicated CLTI.

Cases (*n* = 42)	CLTI + SSc	Uncomplicated CLTI	*P* value
	*n* = 7	*n* = 35	
Sex (M:F)	1:6	22:13	<0.05
Age (years)	61–79 (70)	56–91 (75)	0.117
Follow‐up time (months)	1–70 (27.4)	1–129 (29.8)	
Medical history			
Hypertension	5	28	0.614
Dyslipidemia	4	19	0.89
Chronic renal failure	1	19	0.053
Diabetes	1	19	<0.05
Heart failure	5	23	0.355
Smoking history	4	21	0.888
Medical treatment			
Antibiotics	6	22	0.242
Intravascular treatment	6	32	0.638
Amputation	2	13	0.522

Abbreviations: CLTI, chronic limb‐threatening ischemia; F, female; M, male. SSc, systemic sclerosis.

**FIGURE 1:**
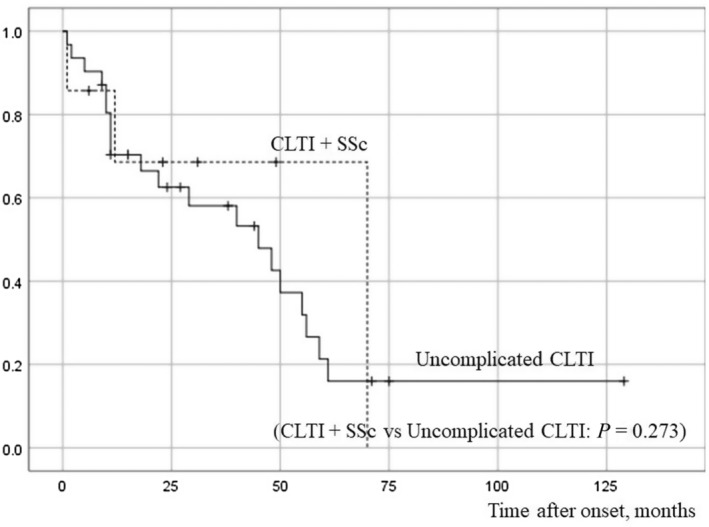
The follow‐up time from the onset of ulceration in cases with CLTI and systemic sclerosis (SSc) and in those with uncomplicated chronic limb‐threatening ischemia (CLTI). There were no significant differences in survival times between cases with CLTI and SSc versus uncomplicated CLTI cases.

Of the 42 cases enrolled in our study, a follow‐up study was possible in a total of 38 patients; the median times to follow‐up were 23 months (1 to 70 months) in patients with CLTI and SSc, and 22 months (1 to 129 months) in patients with uncomplicated CLTI, indicating no statistically significant difference (Figure 1).

The median survival time from the onset of ulceration was 70.0 months in CLTI and SSc, and 45.0 months in uncomplicated CLTI, indicating no significant difference in prognosis between them (*P =* 0.273). During the follow‐up period, three of seven patients (42.9%) with CLTI and SSc died, and 20 of 31 (65.0%) with uncomplicated CLTI died (*P* = 0.52). The causes of death of patients with CLTI and SSc were sepsis (case 6) and congestive heart failure (case 3).

We also analyzed the characteristics of the patients with CLTI and SSc (Table 2). Among the seven patients, two exhibited diffuse cutaneous SSc while five had limited cutaneous SSc. These patients mostly presented with toe ulcers. EVT was administered to six of the seven patients, with resultant ulcer healing in two cases (cases 5 and 7). Amputation was performed in two patients after revascularization due to severe pain in case 1 and persistent osteomyelitis in case 2. Case 3 died due to sepsis without EVT.

**TABLE 2:** Characteristics and outcomes of the seven CLTI and SSc cases.

	1	2	3	4	5	6	7
SSc	dc	lc	lc	lc	lc	dc	lc
Antinuclear antibody	ACA(+), Scl70(+)	ACA(+)	ACA(+)	ACA(+)	ACA(+)	Scl70(+)	ACA(+)
Sex	F	F	F	F	F	F	M
Onset age (years)	74	79	72	66	61	68	70
Duration from diagnosis of SSc to ulcer onset	18	7	3	32	0	7	3
Disease duration of ulcer (months)	14(Lt), 41(Rt)	6	12	24	1	1	3
Skin symptoms	Lt. 1st toe; Rt. 1st and 5th toe ulcer	Rt. plantar ulcer and necrosis	Lt. 1st toe necrosis	Bilateral internal ankle ulcer	Lt. 1st toe ulcer	Rt. plantar skin necrosis	Rt. 1st toe ulcer
Complications							
Lung symptoms	IP	–	Bacterial pneumonia	PH	–	–	PH
Renal symptoms	–	CKD	–	–	–	–	–
Antiphospholipid syndrome	–	–	–	–	–	–	–
Past history	DM, MS, AS, HT	DCM, AP, HT	OMI	AS, Af	GERD	RA, IP, MS, HT	OMI, HT, HL
ABI (lt./rt.)	0.91/ND	ND	ND	1.18/1.23	0.73/ND	0.87/1.14	1.12/1.21
SPP (lt./rt. dorsal‐plantar)	20–46/34–35	NA	43–39/50–10	40–51/28–42	18‐38/ND	ND	ND
Drug treatment of SSc							
Prednisolone (mg)	–	–	10	4	–	7	–
Bosentan	〇	–	–	–	–	–	〇
Ca blocker	–	–	–	–	–	〇	–
Antiplatelet agent	–	〇	–	〇	〇	–	〇
Prostaglandin drug	–	〇	〇	–	–	〇	〇
Other main Treatment	EVT (Lt. POPA), heparinization, blood purification (Rheocarna®)	EVT (rt. ATA)	External preparation only	EVT (lt.PTA), HBO	EVT (lt. EIA)	EVT (rt. POPA)	EVT (rt. foot dorsal artery)
Outcome	Amputation (lt. leg), alive	Amputation, alive	Dry necrosis, dead (12 M later)	No change	No skin lesion, alive	Sepsis, dead (1 M later)	No skin lesion, alive

Abbreviations: ABI, ankle‐brachial index; ACA, anticentromere antibody; Af, atrial fibrillation; AP, angina pectoris; AS, aortic stenosis; ATA, anterior tibial artery; CKD, chronic kidney disease; CLTI, chronic limb‐threatening ischemia; dc, diffuse cutaneous SSc; DCM, dilated cardiomyopathy; DM, diabetes mellitus; EIA, external iliac artery; EVT, endovascular therapy; F, female; GERD, gastroesophageal reflux disease; HBO, hyperbaric oxygen therapy; HL, hyperlipidemia; HT, hypertension; IP, interstitial pneumonia; lc, limited cutaneous SSc; lt, left; M, months; M, male; MS, mitral stenosis; NA, not available; ND, not done; OMI, old myocardial infarction; PH, pulmonary hypertension; POPA, popliteal artery; PTA, posterior tibial artery; RA, rheumatoid arthritis; rt, right; Scl70, anti‐Scl70 antibodies; SPP, skin perfusion pressure; SSc, systemic sclerosis.

‘〇’ means these patients have been treated with these drugs.

## 4 DISCUSSION

The management of CLTI ulcer requires consideration of various factors, such as wound site, size, presence of infection and ischemia, all of which contribute to the risk of lower extremity amputation. The CLTI risk estimates include old age, CRF, coronary artery disease, congestive heart failure, diabetes, smoking, and cerebrovascular diseases.^3^ A review article of untreated CLTI, which included 13 studies and 1527 patients, showed a mortality rate of CLTI of 22% and major amputation rate of 22%.^5^

The patients with SSc showed a significant high risk of peripheral arterial disease.^6^ The association of the patients with SSc and a lower limb amputation were reported that pulmonary artery hypertension, smoking, and corticosteroid use.^7^ In our study, cases with CLTI and SSc exhibited a significantly lower prevalence of diabetes and male‐to female ratio compared to uncomplicated CLTI (*P* < 0.05). However, no other significant differences were observed in patient background characteristics, treatment options (Table 1), and median survival time between them (Figure 1). These could be related to (1) small sample size, (2) diabetes and CRF being important risk factors for uncomplicated CLTI, and (3) five of the seven patients with CLTI and SSc presented with limited cutaneous scleroderma, which does not present with severe systemic symptoms.

In our study, major or minor amputations were performed after revascularization in two of the seven CLTI and SSc cases, and there was no significant difference in the proportion of patients who underwent amputation between the two groups (*P* = 0.522; Table 1). Both are still alive and have experienced no pain or recurrence of ulcers (Table 2). There has been a report of five cases with SSc incurable skin ulcers that were successfully cured by amputation.^2^ These reports and our data suggest that amputation should be considered only after achieving adequate infection control, analgesic management, and optimizing blood flow to alleviate pain, whereas Hasegawa says major amputation should be avoided as much as possible in the treatment of SSc‐related limb issues, since the amputation sites are prone to suture failure and recurrence of ulcers postoperatively.^8^ However, further research is needed to evaluate the efficacy of amputation in patients with SSc because of the small sample size in this study.

The opinion on endovascular treatment for scleroderma is divided due to reported complications such as severe vasospasm and thrombosis.^9^, ^10^ While there are risks associated with vascular treatment in SSc, studies indicate favorable outcomes, with amputation‐free survival rates reaching 89% at 1 year post‐EVT for connective tissue diseases and CLTI.^11^ Deguchi et al. reported that six of eight patients with SSc treated with bypass surgery achieved pain relief and initial wound healing.^12^ However, the long‐term outcomes in 18 SSc patients after open infra‐inguinal bypass surgery were significantly worse than those in 410 non‐SSc patients due to graft failure, and the limb salvage rate was 72%.^13^ These results could be related to the development of structural vascular changes in SSc, such as destructive and proliferative obliterative vasculopathy.^14^ Among CLTI and SSc in our study, EVT was performed in six of the seven patients, while amputation was performed in two patients. Among those who underwent EVT, 33% (2/6 cases, cases 5 and 7) achieved epithelialization and 67% (4/6 cases, cases 1, 5, 6 and 7) experienced pain relief (Table 2).However, we need more investigation due to the low sample numbers, and performance of EVT should be a collaborative decision with specialists in the other involved department, taking into account factors such as infection, pain, blood flow, and the patient's general physical condition.

Our results suggest that revascularization might be a viable treatment option for CLTI and SSc patients experiencing severe pain refractory to conservative measures.

### REPRINT REQUESTS

Yoshihiro Matsuda.

## CONFLICT OF INTEREST STATEMENT

Shin Morizane is an Editorial Board member of The Journal of Dermatology and a co‐author of this article. To minimize bias, he was excluded from all editorial decision‐making related to the acceptance of this article for publication. Except for this, none declared.
